# The effect of a person-centred lifestyle programme on cancer-related fatigue in colorectal cancer survivors: a randomised trial

**DOI:** 10.1017/S0007114525105862

**Published:** 2026-02-14

**Authors:** Judith de Vries-ten Have, Laura H. H. Winkens, Auke J. C. F. Verkaar, Sharon A. G. Bloemhof, Lara Schepers, Koen Manusama, Sandra Beijer, Dirkje W. Sommeijer, Ramon R. J. P. van Eekeren, Flip Kruyt, Alinda Guitink, Ellen Kampman, Renate M. Winkels

**Affiliations:** 1 Chair Group Nutrition and Disease, Division of Human Nutrition and Health, Wageningen University and Research, Wageningen, The Netherlands; 2 Consumption and Healthy Lifestyles Chair Group, Wageningen University and Research, Wageningen, The Netherlands; 3 Department of Research & Development, Netherlands Comprehensive Cancer Organisation (IKNL), Utrecht, The Netherlands; 4 Department of Dietetics, Maastricht University Medical Center+, Maastricht, The Netherlands; 5 Department of Internal Medicine, Flevohospital, Almere, The Netherlands; 6 Department of Surgery, Rijnstate Hospital, Arnhem, The Netherlands; 7 Department of Surgery, Hospital Gelderse Vallei, Ede, The Netherlands; 8 Department of Gastroenterology, Deventer Hospital, Deventer, The Netherlands

**Keywords:** Cancer-related fatigue, Intervention, Diet, Physical activity, Personalisation, Lifestyle, Behaviour change

## Abstract

Cancer-related fatigue is a common problem among colorectal cancer (CRC) survivors even after completion of treatment. In a randomised trial, we assessed the effect of a person-centred lifestyle programme on cancer-related fatigue among CRC survivors who completed treatment. Survivors who completed treatment at least 6 months but no longer than 5 years ago and who were experiencing cancer-related fatigue were randomised to intervention or control group. The intervention group worked with a lifestyle coach for 6 months during twelve sessions to stepwise increase adherence to the World Cancer Research Fund/American Institute of Cancer Research cancer prevention guidelines on healthy diet and physical activity. The control group did not receive lifestyle coaching. Changes in cancer-related fatigue from baseline to 6 months were assessed with the FACIT (Functional Assessment of Chronic Illness Therapy) – Fatigue Scale. As a secondary outcome, we assessed changes in health-related quality of life (HRQoL). Higher scores indicate less fatigue and better HRQoL. Eighty participants were randomised to the intervention group; eighty-one to the control group. Baseline characteristics were similar: mean age 64·1 (sd 10·9) years; 55·3 % were women; and 72 % had colon cancer. There were favourable changes in dietary behaviours and physical activity in the intervention group; the control group did not show changes to the same extent. The programme did not result in statistically significant differential changes over time between intervention and control group in cancer-related fatigue (0·8; 95 % CI −1·6, 3·2) or HRQoL (1·3; 95 % CI −2·2, 4·8). A person-centred lifestyle programme improved the lifestyle of CRC survivors, but the programme was not effective in reducing cancer-related fatigue or in improving HRQoL.

Many colorectal cancer (CRC) survivors who completed treatment continue to experience symptoms related to the disease and/or treatment; those symptoms can have a large impact on their health-related quality of life (HRQoL)^(1)^. Cancer-related fatigue (hereafter referred to as ‘fatigue’) is among the most reported and severe problems during CRC survivorship^(1)^. The prevalence of fatigue in the first 5 years after diagnosis ranges from roughly 40 % to 70 %, depending on sociodemographic (e.g. sex and age) and clinical (e.g. treatment and number of co-morbidities) characteristics^(2–4)^. Fatigue can persist many years after diagnosis, as shown by a registry-based study conducted in France that reported the prevalence of fatigue to be 45 % in CRC survivors 15 years after diagnosis^(5)^.

Fatigue is a multifactorial symptom and can be influenced by demographic, medical, psychosocial, behavioural and biological factors^(6)^. Currently, there is no golden standard for the treatment of fatigue^(6)^. Observational data suggest that having a healthier lifestyle, including the consumption of a healthy diet and being sufficiently physically active, is associated with less fatigue after completion of cancer treatment in CRC survivors^(7–9)^.

Recently, we reviewed and summarised the literature on lifestyle interventions that targeted fatigue among cancer survivors who finished treatment^(10)^, where lifestyle interventions were defined as either physical activity and/or nutrition-related interventions. The review showed that only three out of twenty-nine randomised controlled trials specifically targeted CRC survivors^(11–13)^. CRC survivors may have different factors that influence healthy eating, physical activity and/or exercise than other cancer survivors, as CRC survivors may have a stoma and/or bowel dysfunction, which requires tailoring of the intervention to specifically CRC survivors^(14)^. Three additional lifestyle studies with CRC survivors^(15–17)^ were identified that were not included in our review^(10)^, as those studies also included survivors who were still receiving adjuvant treatment, while our review focused on studies that included survivors who had completed treatment.

One of these six previously conducted trials with CRC survivors took both physical activity and nutrition into account^(17)^, whereas the other studies focused only on physical activity^(11–13,15)^ or yoga^(16)^. None of these trials showed statistically significant differences in changes in fatigue over time between the intervention group(s) and control group^(11–13,15–17)^. Important to state is that none of the six trials recruited participants based on their level of fatigue, making it difficult to detect improvements in fatigue as not all participants experienced fatigue^(6,10)^. Thus, there is a clear need for a randomised controlled trial that investigates the effectiveness of a combined diet and physical activity lifestyle intervention on fatigue among CRC survivors who completed treatment and who experience fatigue.

This randomised controlled trial was conducted with the primary aim to test the effect of a 6-month person-centred lifestyle intervention including both diet and physical activity on fatigue in CRC survivors who completed treatment and who experience fatigue. Since fatigue is rarely an isolated symptom, the effect of the lifestyle intervention on HRQoL was examined as a secondary outcome.

## Methods

### Study design

The study was called the SoFiT study and was a two-arm parallel randomised controlled trial with an intervention group and a control group. Details of the design of the study have been previously described^(18)^. The study was approved by a Medical Research Ethics Committee (CMO region Arnhem-Nijmegen, NL75999.091.21, METC nr 2021-8182). All participants provided written informed consent.

### Eligibility criteria

Persons were eligible when they were adult CRC survivors who completed CRC treatment for stage I–III disease at least 6 months but no longer than 5 years ago and were experiencing fatigue according to the Functional Assessment of Chronic Illness Therapy (FACIT) – Fatigue Scale^(19)^. A score lower than 34 was defined as experiencing fatigue, as this was previously identified as a cut-off point for the diagnosis of cancer-related fatigue^(20)^. Additional inclusion criteria were that persons had to live within a reasonable distance from the research centre (within approximately 1·5 h of driving by car from Wageningen University & Research), had to be willing to be randomised into either the intervention or control group and had to be able to speak, write and read Dutch. Persons were not eligible when they participated in another study that could interfere with the current study, had chronic drug abuse and excessive alcohol consumption (more than four glasses/d on average), or were unwilling or unable to comply with the intervention (e.g. through dementia or severe mental illness).

### Recruitment and screening

Three different recruitment routes were used: (1) recruitment through regional hospitals, (2) recruitment through the ‘Prospectief Landelijk’ CRC cohort (PLCRC, Prospective National CRC cohort)^(21,22)^ and (3) recruitment through local and social media. Persons who expressed interest in participation through any of these routes were screened for eligibility using an online (or paper) questionnaire through Castor Electronic Data Capture (Castor EDC). The questionnaire consisted of the FACIT-Fatigue Scale and a questionnaire to assess the other inclusion and exclusion criteria.

### Randomisation and blinding

The study team visited participants at the participants’ homes for baseline data collection. To avoid bias in baseline data due to group allocation, participants were randomised to the intervention or control group at the end of this home visit. A stratified block randomisation procedure was used with permuted blocks of varying block sizes (4, 6 and 8). We stratified for the level of fatigue (extreme fatigue ≤ 20, or fatigue > 20, as determined by the FACIT-Fatigue Scale) and for chemotherapy as part of the treatment (self-reported chemotherapy: yes/no) and used the randomisation module of Castor EDC for the randomisation process. The nature of the intervention did not allow us to blind participants to group allocation as participants were aware whether they were in the intervention or control group. Assigned participant IDs were pseudo-anonymised. An independent researcher assigned random letters to the treatment groups, concealing which group was the intervention group and which was the control group, to ensure that data analysis was conducted in a blinded manner.

### Intervention group

Participants randomised to the intervention group received a 6-month lifestyle programme. The design and content of the lifestyle programme have been previously described in detail^(18)^. The lifestyle programme focused on lifestyle improvements that can be maintained over the long term to achieve sustained behaviour change. Each participant worked with one of two lifestyle coaches for 6 months to gradually increase adherence to the World Cancer Research Fund cancer prevention guidelines on healthy diet and physical activity^(23)^. The lifestyle coach contacted each participant every 2 weeks, for a total of twelve appointments, over the course of 6 months. This included four home visits and eight telephone or video calls. Participants received a handbook and brochures to assist in making lifestyle changes. The programme adopted a person-centred approach by (1) tailoring to the participant’s current lifestyle and personal characteristics, (2) targeting personal behavioural determinants and (3) considering the participant’s preferences, opportunities and disease-related barriers^(18)^. The lifestyle coaches applied behaviour change techniques tailored to each participant and session^(18)^.

### Control group

The control group did not receive lifestyle coaching sessions during the 6-month period. To promote retention, participants in the control group received newsletters after 1·5 and 4·5 months with content unrelated to lifestyle behaviour. At 3 months, they received a small incentive, a postcard, a questionnaire and a phone call as part of the data collection. To promote retention, we also informed participants in the control group that they were entitled to receive two lifestyle coaching sessions and the same lifestyle information materials that the intervention group received after completion of the 6-month study period. The control group continued to receive their care as usual by their treating physicians, which may include survivorship care.

### Data collection

Data were collected via online questionnaires and during home visits at baseline and at 6 months. Upon request, participants could receive paper versions of questionnaires, which the research team entered in Castor EDC. All data were entered and collected via Castor EDC.

### Description of anthropometrics, dietary intake and physical activity

We assessed anthropometrics, dietary intake and physical activity to describe lifestyle behaviour at baseline and 6 months. Anthropometrics included body weight, height and waist circumference. During the home visits, researchers measured participants’ body weight with a calibrated scale, waist circumference with a tape measure and height with a stadiometer.

To assess habitual dietary intake at baseline and 6 months, participants completed a semi-quantitative FFQ. This FFQ was designed and validated by the Division of Human Nutrition and Health of Wageningen University^(24,25)^. The FFQ included eighty-five questions about food items. Participants reported the intake of foods and drinks consumed during the previous month; frequencies of intake were combined with standard portion sizes and household measures to quantify intake. Nutritional intake of fibre and alcohol was calculated by linking food and drink items according to the Dutch National Food Consumption Tables (NEVO 2010). The FFQ was used to quantify dietary intake in g/d, or glasses per week for the alcohol guideline. For the guideline on ‘fast foods’ and other processed foods, we took all foods and drinks into account that were high in fat, starches and/or sugars (e.g. (fried) foods, cookies, sweets and sauces). For the guideline on red and processed meat, we made the distinction between red meat and processed meat, where we included processed *red* meat as processed meat. Each food or drink item was assigned to only one food group.

Physical activity was measured both subjectively and objectively with a questionnaire and an accelerometer at baseline and 6 months. For the subjective measurement of physical activity, the Short Questionnaire to Assess Health (SQUASH)-enhancing physical activity^(26)^ was used. The questions in the SQUASH were asked during the home visits and were pre-structured in commuting, work/school activities, household activities, leisure time and sports. The two questions in the SQUASH survey, ‘days per week’ and ‘average time per day’, were used to estimate physical activity of a usual week in the past months. Scores were assigned to the different reported activities based on intensities in metabolic equivalent (MET) according to a previously published compendium^(27)^. This was translated to minutes of moderate and vigorous physical activity (MVPA) per week. Activities were scored as MVPA when they had a MET score of ≥ 3. We also estimated the number of days participants did muscle- and bone-enhancing activities using the classification of the physical activity guidelines report of the Dutch National Institute for Public Health and the Environment^(28)^. In that report, bone-enhancing activities are defined as strength training, or as activities that involve bearing the body’s own weight (e.g. walking, boxing and tennis), while muscle-enhancing activities are defined as activities that focus on endurance (e.g. cycling, swimming and fitness)^(28)^. All activities that are bone-strengthening are also considered to be muscle-strengthening. Some activities are only muscle-strengthening and not bone-strengthening, and there are no activities that are muscle-strengthening but not bone-strengthening^(28)^.

For the objective measurement, MVPA (i.e. activities with MET ≥ 3 in min/week) was assessed by placing an accelerometer, the activPAL3 micro (PAL Technologies Ltd, Glasgow, UK), on the upper thigh and wearing it for 24 h a day during nine consecutive days. During the baseline house visit, the accelerometer was placed by the researcher. At the end of the intervention, participants received the accelerometer with placement instructions via post. Participants placed the accelerometer themselves and wore it during the 9 d before the house visit, during which it was collected by the researcher. Raw accelerometer data were processed with PAL analysis software (PAL Software Suite, version 8, PAL Technologies) and analysed using a script based on the algorithm of Winkler *et al.* ^(29)^. The accelerometer was worn for a median of eight valid days at baseline (interquartile range 7–8) and for a median of seven valid days at the end of the study (interquartile range 6–7). At baseline, nine persons had less than four valid days, and at 6 months, eleven persons had less than four valid days. The number of valid days was similar across timepoints and groups.

### Primary outcome: cancer-related fatigue

Fatigue was assessed during screening to assess eligibility and was again assessed at baseline and 6 months with the thirteen-item FACIT-Fatigue Scale, which is a widely used, validated, reliable and recommended questionnaire^(30,31)^. Each item is scored with a five-point Likert scale, which results in a score that ranges from 0 to 52^(19)^. Lower scores indicate higher fatigue levels.

### Secondary outcome: health-related quality of life

As fatigue is rarely an isolated symptom, we also assessed whether HRQoL changed during the 6 months of the study. The thirty-six-item Functional Assessment of Cancer Therapy – Colorectal (FACT-C) questionnaire was used for this assessment at baseline and 6 months^(32)^. Of these thirty-six items, thirty-four items are used to calculate a score ranging from 0 to 136; the remaining two items are only relevant for persons with an ostomy but were not used in the current study. HRQoL is assessed with the following domains: physical well-being, social/family well-being, emotional well-being, functional well-being and CRC subscale^(32)^. Higher scores indicate better HRQoL.

### Other study parameters: baseline characteristics

Sociodemographic information (i.e. age, sex, living situation, education and employment), smoking status and number of co-morbidities were self-reported and collected with questionnaires at baseline. The clinical parameters time since last treatment, tumour location, stage of disease and information on cancer treatment were obtained via the Netherlands Cancer Registry managed by the Netherlands Comprehensive Cancer Organisation (IKNL). Information on whether participants had a stoma was collected by the FACT-C CRC subscale.

### Data analysis

An estimated sample size of 184 participants was previously calculated to provide 80 % power to detect an effect on the primary outcome. In this calculation, we considered that a three-point differential change between groups would be a clinically important difference with an sd of 6·7 and accounted for 15 % drop-out^(18)^. Due to an ending of grant funding, we did not reach the intended sample size and had to stop recruitment after including 161 participants, of which fourteen participants dropped out (see results for further information). This means we had 77 % power in this study.

To test the effect of the lifestyle intervention on the primary outcome – fatigue – an ANCOVA was performed. In the ANCOVA analysis, fatigue at 6 months was compared between intervention and control group while adjusting for fatigue at baseline. Additionally, the stratification factors used during randomisation (i.e. extreme fatigue ≤ 20, or fatigue > 20 on the FACIT-Fatigue Scale and chemotherapy yes or no) were included in the ANCOVA model^(33)^. The primary analysis was conducted on an intention-to-treat basis.

Missing data for fatigue at 6 months (9·3 %) were imputed using multiple imputation. There were no missing data for fatigue at baseline. Missing data at 6 months occurred because participants did not complete the questionnaires, dropped out of the study and/or were taken out of the study by the study team due to recurrence or development of a second primary cancer during the study. Multiple imputation by chained equations (MICE), a fully conditional specification method, was conducted using the ‘mice’ package for R software, drafting ten datasets and using ten iterations^(34)^. In the imputation process, we used a correlation matrix to determine which predictors should be added: predictors with a correlation higher than 0·1 or lower than –0·1 with the outcome were added. In case of a high correlation between predictors (higher than 0·7/lower than –0·7), we only added the predictor with the highest correlation with the outcome.

Two sensitivity analyses were conducted to assess the robustness of the effect on the primary outcome. First, a complete case analysis was conducted, which included only those participants who had complete data on fatigue at baseline and follow-up. Second, a per-protocol analysis was conducted, in which the participants in the intervention group who were adherent to the protocol were compared with the control group. Per protocol was defined as ‘attended at least 11 out of the 12 scheduled coaching sessions’. Missing data for fatigue for the per-protocol analysis were imputed in the same manner as for the primary analysis.

The secondary outcome, HRQoL, was analysed in the same manner as the primary outcome by using a series of ANCOVA models. Missing data were imputed for total HRQoL and subdomains at baseline (0–0·6 %) and at 6 months (9·3–10·6 %). We reported the total HRQoL score and scores on the separate domains.

After conducting all analyses according to the pre-specified data analysis plan, we decided to add the following three analyses to the results of this paper. The first *post hoc* analysis included those participants who were classified as ‘experiencing fatigue’ (score of < 34 on the FACIT-Fatigue Scale) according to their baseline data. Participants were eligible for the trial when they were experiencing fatigue during screening. Nevertheless, fatigue was reassessed at baseline; there were roughly 4 weeks between the screening and the baseline visit. The result of that baseline assessment differed from the assessment of fatigue during screening/eligibility testing.

The second *post hoc* analysis included only participants who had received chemotherapy as part of their treatment, as fatigue levels might be higher in CRC survivors who underwent chemotherapy^(4,35)^, potentially leaving more room for improvement.

The third *post hoc* analysis included only participants who only had surgery as part of their treatment. All *post hoc* analyses were conducted in the same manner as the primary analysis. R Studio version 4.3.1 was used for the analyses, and a two-sided alpha of 0·05 was employed.

## Results

### Characteristics of the study population

**Fig. 1.**
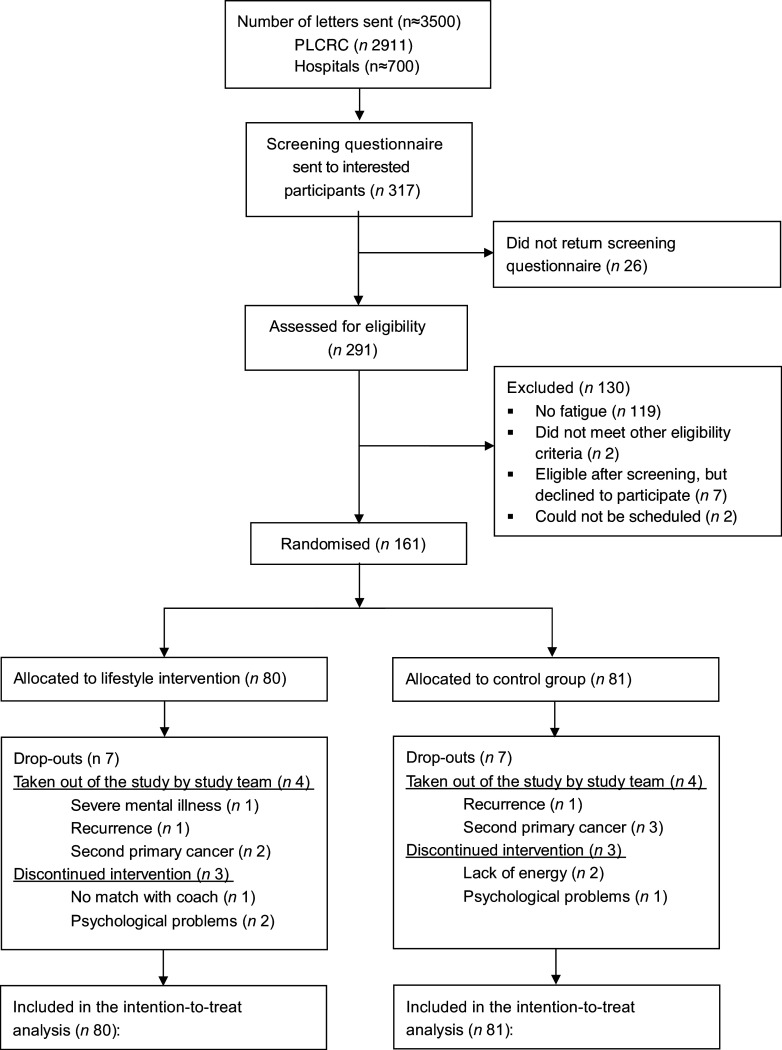
Flow chart of participants included in the randomised controlled trial and in the analyses of the primary and secondary outcomes. PLCRC, Prospectief Landelijk CRC Cohort.

In total, 317 persons showed interest in the study and received a screening questionnaire. This was returned by 291 persons, of which 161 participants were eligible and included in the study; see Figure 1. The primary reason for ineligibility was not being classified as experiencing fatigue according to the FACIT-Fatigue Scale. Of the eligible 161 participants, eighty were randomised to the intervention group and eighty-one were randomised to the control group. Fourteen participants (8·7 %), seven in each group, were excluded or dropped out of the study. Reasons for exclusion or drop-out were psychological problems, development of second primary cancer, or a recurrence, lack of energy to continue with the study, having no match with the lifestyle coach or severe mental illness. The baseline characteristics, including fatigue assessed during screening/recruitment and baseline, and HRQoL, were similar between the intervention and control group (Tables 1, 2 and 3). The mean age of the population was 64·1 (sd 10·9) years, with 55·3 % women, and over half of the population was highly educated (54·7 %). Most participants had colon cancer (72 %), and over half of the participants had stage III disease (54 %). The median time since treatment was 21·8 months, and 46 % of participants had received chemotherapy as part of treatment.

**Table 1.** Baseline characteristics of a randomised controlled trial on lifestyle and fatigue, shown for the total study population and separately for the intervention and control group

Characteristics *n* (%)	Total population		Intervention group		Control group	
	(*n* 161)		(*n* 80)		(*n* 81)	
	Mean	sd	Mean	sd	Mean	sd
Age (years)						
Mean (sd)	64·1	10·9	64·8	11·3	63·3	10·6
Range	39–91		42–88		39–91	
	*n*	%	*n*	%	*n*	%
Sex						
Women	89	55·3	46	57·5	43	53·1
Men	72	44·7	34	42·5	38	46·9
BMI^*^ (kg/m^2^)						
Normal	41	25·6	20	25·0	21	26·2
Overweight	62	38·8	35	43·8	27	33·8
Obese	57	35·6	25	31·2	32	40·0
Education level^†^						
Low	22	13·7	14	17·5	8	9·9
Middle	51	31·7	24	30·0	27	33·3
High	88	54·7	42	52·5	46	56·8
Employment						
Yes	73	45·3	33	41·2	40	49·4
No	88	54·7	47	58·8	41	50·6
Living situation						
Alone	33	20·5	14	17·5	19	23·5
With someone	128	79·5	66	82·5	62	76·5
Smoking status^*^						
Current	5	3·1	3	3·8	2	2·5
Former	84	52·2	38	47·5	46	57·5
Never	71	44·4	39	48·8	32	40·0
Tumour location						
Colon	118	73·3	58	72·5	60	74·1
Rectum	43	26·7	22	27·5	21	25·9
	Median	IQR	Median	IQR	Median	IQR
Time since last treatment, months						
Median (IQR)^*^	21·8	13·2–39·4	23·0	15·0–43·6	19·4	13·0–37·3
	*n*	%	*n*	%	*n*	%
Completed treatment						
Surgery only	78	48·4	40	50·0	38	46·9
Surgery and chemotherapy	55	34·2	26	32·5	29	35·8
Surgery and radiotherapy	9	5·6	4	5·0	5	6·2
Chemotherapy and radiotherapy	6	3·7	3	3·8	3	3·7
Surgery, chemotherapy and radiotherapy	13	8·1	7	8·8	6	7·4
Stage of disease						
I	34	21·1	17	21·2	17	21·0
II	40	24·8	19	23·8	21	25·9
III	87	54·0	44	55·0	43	53·1
Stoma						
Yes	22	13·7	8	10·0	14	17·3
No	139	86·3	72	90·0	67	82·7
Co-morbidities						
0	85	52·8	43	53·8	42	51·9
1	48	29·8	26	32·5	22	27·2
≥ 2	28	17·4	11	13·8	17	21·0
Fatigue						
≤ 20	29	18·0	14	17·5	15	18·5
> 20	132	82·0	66	82·5	66	81·5

IQR, interquartile range.

* Data missing for 1 participant.

† Education was categorised in low (elementary school and secondary education), middle (secondary vocational education) and high (higher professional and university education).

**Table 2.** Between-group differences in changes in fatigue in a randomised controlled study among colorectal cancer survivors

	Intervention group					Control group					Between-group difference^‡^		
	*n*	Baseline		Six months		*n*	Baseline		Six months		*n*	Adj. mean difference	95 % CI
		Mean	sd	Mean	sd		Mean	sd	Mean	sd			
Intention-to-treat													
Fatigue^†^	80	28·1	7·3	34·2	9·4	81	28·4	7·0	33·5	8·3	161	0·8	–1·6, 3·2
Complete cases													
Fatigue	72	28·4	7·3	34·5	9·4	74	28·3	7·0	33·8	8·3	146	0·6	–1·9, 3·0
Per protocol													
Fatigue	62	28·6	6·7	35·5	8·9	81	28·4	7·0	33·5	8·3	143	1·8	–0·7, 4·2

† Fatigue was measured with the Functional Assessment of Chronic Illness Therapy (FACIT) – Fatigue Scale with score range 0–52.

‡ Between-group mean differences were adjusted for fatigue at baseline and for the stratification factors used during randomisation. Missing data were imputed for the intention-to-treat and per-protocol analysis. For the complete case analysis, data were analysed for participants who had complete data on fatigue at baseline and at 6 months. Per protocol was defined as ‘attended at least 11 out of the 12 scheduled coaching sessions’.

**Table 3.** Between-group differences in changes in health-related quality of life in a randomised controlled study among colorectal cancer survivors

	Intervention group					Control group					Between-group difference^‡^		
	*n*	Baseline		Six months		*n*	Baseline		Six months		*n*	Adj. mean difference	95 % CI
		Mean	sd	Mean	sd		Mean	sd	Mean	sd			
Intention-to-treat													
HRQoL total^†^	80	94·3	13·2	100·4	16·1	81	95·0	15·4	99·8	15·4	161	1·3	–2·2, 4·8
Physical well-being	80	21·1	3·0	22·6	3·7	81	21·1	3·1	22·7	3·3	161	–0·1	–1·0, 0·9
Social well-being	80	19·1	4·8	19·4	4·7	81	18·7	5·4	18·8	5·3	161	0·3	–0·8, 1·3
Emotional well-being	80	18·0	3·5	19·3	3·4	81	18·5	3·6	19·6	3·3	161	0·0	–0·8, 0·9
Functional well-being	80	15·7	4·4	17·7	5·1	81	16·1	4·3	17·5	4·5	161	0·5	–0·7, 1·8
Colorectal cancer subscale	80	20·4	3·6	21·4	4·1	81	20·7	4·2	21·2	3·9	161	0·3	–0·7, 1·3
Complete cases													
HRQoL total	72	94·1	13·5	100·5	16·4	72	94·8	16·1	99·9	15·7	144	1·1	–2·4, 4·6
Physical well-being	72	21·1	2·9	22·7	3·7	74	21·1	3·1	22·8	3·2	146	–0·2	–1·1, 0·8
Social well-being	72	19·1	4·8	19·4	4·7	74	18·8	5·5	18·9	5·3	146	0·3	–0·8, 1·3
Emotional well-being	72	18·1	3·5	19·3	3·5	73	18·5	3·6	19·6	3·3	145	–0·0	–0·9, 0·9
Functional well-being	72	15·6	4·5	17·7	5·1	74	16·0	4·4	17·4	4·5	146	0·6	–0·6, 1·8
Colorectal cancer subscale	72	20·2	3·6	21·4	4·1	72	20·5	4·3	21·3	3·9	144	0·2	–0·8, 1·2
Per protocol													
HRQoL total	62	94·6	12·9	101·7	15·5	81	95·0	15·4	99·8	15·4	143	2·2	–1·5, 5·9
Physical well-being	62	21·1	2·8	23·0	3·4	81	21·1	3·1	22·7	3·3	143	0·2	–0·7, 1·2
Social well-being	62	19·2	4·4	19·6	4·5	81	18·7	5·4	18·9	5·3	143	0·4	–0·7, 1·4
Emotional well-being	62	18·1	3·2	19·6	3·2	81	18·5	3·6	19·6	3·3	143	0·2	–0·7, 1·1
Functional well-being	62	15·6	4·5	17·9	4·9	81	16·1	4·3	17·5	4·5	143	0·7	–0·5, 2·0
Colorectal cancer subscale	62	20·5	3·3	21·6	4·0	81	20·7	4·2	21·2	3·9	143	0·4	–0·7, 1·5

HRQoL, health-related quality of life.

† HRQoL, assessed with the Functional Assessment of Cancer Therapy (FACT) – Colorectal (FACT-C) questionnaire with score range 0–136. Subscales range from 0 to 28 for physical, social/family and functional well-being and the colorectal cancer subscale, and from 0 to 24 for the emotional well-being subscale.

‡ Between-group mean differences were adjusted for HRQoL at baseline and for the stratification factors used during randomisation. Missing data were imputed for the intention-to-treat and per-protocol analysis. For the complete case analysis, data were analysed for participants who had complete data on HRQoL at baseline and at 6 months. Per protocol was defined as ‘attended at least 11 out of the 12 scheduled coaching sessions’.

### Anthropometrics, dietary intake and physical activity

Over time, the intervention group reported increases in the consumption of fruit and vegetables and decreases in the consumption of processed meat and sugar-sweetened beverages (Table 4). In the control group, most food groups appeared to remain relatively stable over time. Although both groups reported reduced fast-food intake, this reduction seems more pronounced in the intervention group. BMI and waist circumference appeared relatively stable over time in both groups (Table 4). In the intervention group, MVPA appeared to increase in the intervention group and not in the control group according to data of the SQUASH. The accelerometer data suggested an increase in MVPA in the intervention and a decrease in the control group. The control group reported an increase in bone- and muscle-enhancing activities as assessed by the SQUASH, while these activities appeared to remain similar over time in the intervention group. Walking was the most predominant bone- and muscle-enhancing activity that was conducted by the participants. After leaving out ‘walking’ from this category, the median number of bone- and muscle-enhancing activities was zero across timepoints and groups.

**Table 4.** Adherence to the World Cancer Research Fund cancer prevention recommendations at baseline and 6 months, shown for the intervention and for the control group

World Cancer Research Fund recommendation	Operationalisation	Intervention group (*n* 80)						Control group (*n* 81)					
		Baseline			Six months			Baseline			Six months		
		*n*	Median	IQR	*n*	Median	IQR	*n*	Median	IQR	*n*	Median	IQR
1. Be a healthy weight	BMI (kg/m^2^)	80	27·3	25·3–31·0	73	26·7	24·2–30·6	80	28·2	24·9–32·2	73	27·7	24·8–31·4
	Waist circumference (cm)	79	103·8	90·5–111·2	72	98·4	89·2–110·6	80	100·3	88·2–114·7	74	99·9	91·9–110·4
	Men	33	109·0	102·5–116·2	28	109·9	97·5–115·4	37	109·8	98·8–119·2	34	105·9	99·5–115·9
	Women	46	93·9	86·0–105·0	44	92·1	86·3–105·2	43	92·0	85·2–113·0	40	94·9	87·1–106·4
2. Eat a diet rich in whole grains, vegetables, fruit and beans	Consumption of fruit (g/d)	77	158	62–226	70	217	184–240	80	122	68–216	69	126	69–224
	Consumption of vegetables (g/d)	77	137	81–203	70	172	123–245	80	120	61–196	69	131	78–201
	Total fibre intake (g/d)	77	25	18–32	70	23	19–33	80	23	18–28	69	21	17–27
	Beans (g/d)	77	17	11–36	70	22	13–54	80	17	5–35	69	11	0–26
3. Limit consumption of ‘fast foods’ and other processed foods high in fat, starches or sugars^†^	Consumption of foods high in fat, starch and/or sugar (g/d)	77	200	124–264	70	127	82–181	80	209	128–275	69	162	95–261
4. Limit consumption of red meat and processed meat	Red meat (g/d)	77	31	16–52	70	23	11–42	80	27	10–57	69	23	11–43
	Processed meat (g/d)	77	42	19–67	70	14	5–37	80	33	12–60	69	27	9–65
5. Limit consumption of sugar-sweetened drinks	Consumption of sugar-sweetened beverages (g/d)	77	32	7–192	70	20	0–130	80	53	12–175	69	51	7–178
6. Limit alcohol consumption	Consumption of alcoholic beverages (glasses/week)	77	0·3	0·0–0·7	70	0·1	0·0–0·5	80	0·4	0·0–0·9	69	0·4	0·0–1·0
7. Be physically active	Moderate and vigorous physical activity (min/week)												
	SQUASH	80	690	449–1039	74	920	448–1260	81	620	420–940	74	633	473–975
	Accelerometer	72	423	327–550	54	467	350–578	77	427	269–567	48	377	324–526
	Muscle- and bone-enhancing activities (d/week) SQUASH	80	3	1–7	74	3	1–7	81	3	1–7	74	5	2–7

IQR, interquartile ranges; SQUASH, the Short Questionnaire to Assess Health.

† All foods and drinks that were high in fat, starches or sugars were included (e.g. (fried) foods, cookies, sweets and sauces).

### Cancer-Related fatigue

At baseline, the intervention group had a mean fatigue score of 28·1 (sd 7·3), and the control group had a mean fatigue score of 28·4 (sd 7·0) (Table 2). At 6 months, the intervention group showed a slightly greater improvement in fatigue (+6·1) compared with the control group (+5·1). There was no statistically significant differential change in fatigue over time between the intervention and control group (0·8; 95 % CI −1·6, 3·2; Table 2).

### Health-Related quality of life

At baseline, the intervention group had a mean total HRQoL score of 94·3 (sd 13·2) and the control group had a mean total HRQoL score of 95·0 (sd 15·4) (Table 3). At 6 months, the intervention group showed a slightly greater improvement in total HRQoL (+6·1) compared with the control group (+4·8). There was no statistically significant differential change in total HRQoL over time between the intervention and control group (total HRQoL 1·3; 95 % CI −2·2, 4·8) or in any of the subscales (Table 3).

### Additional analyses

#### Sensitivity analyses

Results for the complete case analysis were comparable to the primary analyses. For the per-protocol analyses, we included sixty-two out of eighty participants of the intervention group (77·5 %) as they adhered to the study protocol and completed at least eleven out of twelve lifestyle coaching sessions. The results for the per-protocol analyses were comparable to the primary analyses for fatigue and HRQoL (Tables 2 and 3).

#### *Post hoc* analyses

Upon recruitment, all participants (*n* 161) were classified as experiencing fatigue based on their responses to the recruitment questionnaires. Based on the baseline questionnaire that was administered about 4 weeks after recruitment, the number of participants who were classified as experiencing fatigue was lower (*n* 120) at that timepoint. The first *post hoc* analysis that included only those 120 participants showed comparable results as the primary analyses for fatigue (1·7; 95 % CI −1·1, 4·5), for total HRQoL (1·6; 95 % CI −2·2, 5·4) and for HRQoL subscales (online Supplementary Table S1 and S2). The second *post hoc* analysis that only included those participants who received chemotherapy (*n* 74) showed slightly larger, but statistically non-significant differential changes over time compared with the primary analyses for fatigue (2·3; 95 % CI −1·6, 6·1) and for total HRQoL (2·6; 95 % CI −3·2, 8·4) (online Supplementary Table S1 and S2). The third *post hoc* analysis included participants who only had surgery as part of their CRC treatment (*n* 78). Among those participants, the difference in change in fatigue over time between the groups was 0·3 (95 % CI −3·1, 3·6) and for total HRQoL it was −0·6 (95 % CI −6·0, 4·7) (online Supplementary Table S1 and S2).

## Discussion

We examined the effect of a 6-month person-centred lifestyle intervention on fatigue among CRC survivors who completed treatment and who experienced fatigue. There were favourable changes in dietary behaviours and physical activity in the intervention group, whereas the control group did not show these changes to the same extent. The programme, however, did not result in statistically significant differential changes in fatigue or HRQoL of life between the intervention and control group.

Even though changing lifestyle behaviours may not significantly impact fatigue in CRC survivors who completed treatment, our study demonstrates that it is feasible to improve lifestyle behaviours even among CRC survivors who experience fatigue. This is beneficial as healthy dietary behaviours and sufficient physical activity are associated with lower all-cause mortality and CRC-specific mortality in CRC survivors^(36–39)^. Our finding that changing lifestyle behaviours may not significantly impact fatigue in CRC survivors who completed treatment seems to be supported by findings of earlier conducted comparable trials. We can compare our results to six previous randomised controlled trials of which three were conducted among CRC survivors who had completed treatment^(11–13)^, and three other studies were conducted among CRC survivors who were either undergoing or who had completed treatment^(15–17)^. Similar to our study, those six studies did not find statistically significant changes in fatigue over time between the intervention group(s) and control group^(11–13,15–17)^.

Within groups, we observed a clinically relevant reduction in fatigue of 6·1 points in the intervention group and 5·1 points in the control group, which is almost twice as large as the estimated clinically relevant difference of 3 points for the FACIT-Fatigue Scale^(40)^ and larger than the change reported in previous conducted trials^(11,13,15–17)^. Fatigue scores may have partly improved in both groups due to regression to the mean, but it is unsure how large this effect was. Also, fatigue levels may have naturally improved over time post-treatment^(6,41)^, independent of the study participation. Additionally, fatigue might have improved in both groups due to receiving recognition and attention for the fatigue and cancer journey. The between-group change in fatigue (+0·8) is not clinically relevant; thus, lifestyle changes did not account for the observed improvements in fatigue in both groups. Cancer-related fatigue is a complex symptom and has a multifactorial aetiology^(6)^, for which changing lifestyle may not be sufficient to address all the contributing factors.

Fatigue typically increases during cancer treatment, such as chemotherapy, and typically improves in the year after completion of treatment^(4,6,35)^. Nevertheless, fatigue can be persistent in the years after completion of treatment, which is why we specifically targeted our trial towards persons who were continuing to experience fatigue after completion of treatment. As fatigue is highly prevalent during chemotherapy^(6)^, it can be speculated that survivors who received chemotherapy as part of their cancer treatment are the ones that most likely benefit from a lifestyle intervention after completion of treatment, as fatigue levels may be higher among survivors who had chemotherapy^(6,35)^. In our *post hoc* analysis that included only those participants who received chemotherapy as part of their treatment (*n* 74), we observed that the effect of the lifestyle intervention compared with the control group was more pronounced than in the total study (2·3 points *v*. 0·8 points improvement in the total group). This 2·3 is close to a clinically relevant improvement in fatigue (3 points^(40)^), but it was not statistically significant. Interestingly, baseline levels of fatigue of this subgroup were similar to the fatigue levels of the total population; thus, we speculate that there are other reasons than the severity of fatigue that can explain this trend. Our *post hoc* analysis had a smaller sample size, which reduced the statistical power of this analysis. Given that this was a *post hoc* analysis, results should be interpreted as hypothesis-generating but suggest that studies focusing specifically on those who underwent chemotherapy may be relevant.

Our results suggest that improving lifestyle behaviours may not significantly impact HRQoL in CRC survivors who completed treatment. We observed improvements in total HRQoL for both the intervention group and the control group, but these changes were not statistically different between groups. Our results agree with a meta-analysis on the effect of exercise on HRQoL in CRC survivors after treatment (*n* 379) that also showed no meaningful effects (standard mean difference 0·25; 95 % CI −0·01, 0·51)^(42)^. This supports our findings that lifestyle changes do not result in clinically important improvement in HRQoL in CRC survivors after treatment. As fatigue largely impacts HRQoL^(1,6)^ and the intervention was not effective in improving fatigue, it is not surprising that HRQoL was not significantly improved as well.

Careful consideration of how to quantify behavioural changes is crucial to evaluate actual behaviour change and its impact on the effectiveness of changing fatigue. In this trial, we showed that the intervention group reported substantial changes in dietary and physical activity behaviour. However, accurately quantifying those changes comes with challenges. Dietary intake was reported with an FFQ to give a quantitative ranking of changes in diet. We cannot fully rule out that social desirability in reporting dietary intake and levels of physical activity might have occurred more in the intervention group, since participants in the intervention group received coaching, information and feedback on their lifestyle behaviours during the study by the lifestyle coaches. To reduce the social desirability bias, especially in the intervention group, lifestyle coaches were not involved in data collection of lifestyle behaviours, fatigue and HRQoL. To reduce social desirability bias in capturing changes in specifically physical activity, we used both a subjective and objective measure: a questionnaire to capture physical activity in the last month and an accelerometer to capture physical activity over the last week. Both measurements come with challenges, such as setting thresholds for the accelerometer^(43)^ and over-reporting for questionnaires^(44–46)^. Although those two methods also show different results, they both support our findings that the intervention group appears more active than the control group at 6 months.

### Strengths and limitations

There are several strengths to this study. First, we included only participants who experienced fatigue (i.e. FACIT-Fatigue Scale < 34), in contrast to previous trials^(11–13,15–17)^. If participants are not experiencing substantial fatigue at the start of the study, then not much improvement in fatigue can be expected^(13,17,47)^. Thus, to account for this possibility of ceiling effects of fatigue and as widely recommended in literature^(6,10,13,17,47,48)^, we only included persons who were classified as experiencing fatigue. Second, the drop-out rate was low (8·7 %), while some other studies reported drop-out rates of 15–20 %^(11,16,17)^. Efforts were made for high retention, especially in the control group, such as offering the control group a brief intervention at the end of 6 months, which likely added to the low drop-out^(49)^. Third, we had a good rate of participants complying with attending the scheduled lifestyle sessions (77·5 %).

There are also limitations of this study to consider. First, unlike previous trials^(12,13,16,17)^, we intentionally chose not to select participants based on how healthy their baseline lifestyle was, arguing that every participant could benefit from making changes aligned with the World Cancer Research Fund recommendations, regardless of their starting point. As a result, baseline levels for some of the recommendations were already relatively good (e.g. dietary fibre and alcohol intake). Despite this, we observed evident lifestyle improvements in the intervention group, while the control group did not show changes to the same extent. Second, in similar studies, changes often occur in both the intervention group and control group, as reported by previous trials^(13,15,17)^. Improvements in the control group may contribute to reduced intervention effects. Even though our intervention group made evident lifestyle improvements, and our control group appeared to make fewer changes, we cannot entirely rule out the possibility that changes made by the control group influenced the effects of the intervention. Third, we cannot guarantee that our participants are generalisable to the general CRC survivor population. Less than 10 % of CRC survivors responded to our study invitation letters. Therefore, it could be that we reached a select group of CRC survivors. Among the participants, the percentage of higher education (55 %) was higher than in two cohorts of Dutch CRC survivors (19–35 %)^(50,51)^. The last limitation that we need to address is that we did not fully reach the intended sample size of 184 participants. Due to an ending of grant funding, we had to stop recruitment after including 161 participants. However, this does not limit the validity of the findings. The observed difference in change in fatigue between groups was 0·8, whereas the sample size calculation was based on detecting a difference of 3. This suggests that the actual effect of lifestyle on fatigue is smaller than we originally anticipated. Nevertheless, it is important to acknowledge that a larger number of participants would not necessarily have resulted in ‘statistically significant results’. This modest observed effect is likely reflective of the true impact of lifestyle, rather than a consequence of sample size constraints.

### Conclusion

We demonstrated that a person-centred lifestyle intervention was able to change lifestyle behaviour but was not effective in reducing cancer-related fatigue or in improving HRQoL. Favourable changes were observed in dietary behaviours and physical activity in the intervention group, whereas the control group did not show changes to the same extent. Our results may suggest that lifestyle changes may particularly benefit participants who received chemotherapy, but larger studies are needed to validate these results. The study demonstrates that it is feasible to improve lifestyle behaviours even among CRC survivors who experience fatigue, which is beneficial as a healthy lifestyle is associated with lower all-cause mortality and CRC-specific mortality in CRC survivors.

## Supporting information

de Vries-ten Have et al. supplementary material de Vries-ten Have et al. supplementary material

## Supplementary material

For supplementary material/s referred to in this article, please visit https://doi.org/10.1017/S0007114525105862

## References

[1] Han CJ, Yang GS & Syrjala K (2020) Symptom experiences in colorectal cancer survivors after cancer treatments. Cancer Nurs 43, E132–58. 32000174 10.1097/NCC.0000000000000785 PMC7182500

[2] Adam S, van de Poll-Franse LV, Mols F, et al. (2019) The association of cancer-related fatigue with all-cause mortality of colorectal and endometrial cancer survivors: results from the population-based PROFILES registry. Cancer Med 8, 3227–3236. 31012272 10.1002/cam4.2166 PMC6558477

[3] Drury A, Payne S & Brady AM (2022) Prevalence *v.* impact: a mixed methods study of survivorship issues in colorectal cancer. Qual Life Res 31, 1117–1134. 34417713 10.1007/s11136-021-02975-2 PMC8960628

[4] Husson O, Mols F, van de Poll-Franse LV, et al. (2015) The course of fatigue and its correlates in colorectal cancer survivors: a prospective cohort study of the PROFILES registry. Supportive Care Cancer 23, 3361–3371. 10.1007/s00520-015-2802-x PMC4584107 26123601

[5] Gernier F, Joly F, Klein D, et al. (2020) Cancer-related fatigue among long-term survivors of breast, cervical, and colorectal cancer: a French registry–based controlled study. Supportive Care Cancer 28, 5839–5849. 10.1007/s00520-020-05427-8 32253602

[6] Bower JE (2014) Cancer-related fatigue—mechanisms, risk factors, and treatments. Nat Rev Clin Oncol 11, 597–609. 25113839 10.1038/nrclinonc.2014.127 PMC4664449

[7] Wesselink E, van Baar H, van Zutphen M, et al. (2020) Inflammation is a mediating factor in the association between lifestyle and fatigue in colorectal cancer patients. Cancers 12, 1–13. 10.3390/cancers12123701 PMC7763620 33317113

[8] Eyl-Armbruster RE, Thong MSY, Carr PR, et al. (2022) Change toward healthier lifestyles is associated with better health-related quality of life in long-term colorectal cancer survivors. JNCCN J Natl Compr Cancer Network 20, 1233–1243. 10.6004/jnccn.2022.7049 36351340

[9] van Veen MR, Mols F, Bours MJL, et al. (2019) Adherence to the World Cancer Research Fund/American Institute for Cancer Research recommendations for cancer prevention is associated with better health–related quality of life among long-term colorectal cancer survivors: results of the PROFILES registry. Supportive Care Cancer 27, 4565–4574. 10.1007/s00520-019-04735-y PMC6825038 30927111

[10] de Vries-ten Have J, Winkels RM, Kampman E, et al. (2023) Behaviour change techniques used in lifestyle interventions that aim to reduce cancer-related fatigue in cancer survivors: a systematic review. Int J Behav Nutr Physical Act 20, Article number 126. 10.1186/s12966-023-01524-z PMC10576285 37833784

[11] Kim JY, Lee MK, Lee DH, et al. (2019) Effects of a 12-week home-based exercise program on quality of life, psychological health, and the level of physical activity in colorectal cancer survivors: a randomized controlled trial. Supportive Care Cancer 27, 2933–2940. 10.1007/s00520-018-4588-0 30564936

[12] Brown JC, Damjanov N, Courneya KS, et al. (2018) A randomized dose-response trial of aerobic exercise and health-related quality of life in colon cancer survivors. Psychooncology 27, 1221–1228. 29388275 10.1002/pon.4655 PMC5895514

[13] Pinto BM, Papandonatos GD, Goldstein MG, et al. (2013) Home-based physical activity intervention for colorectal cancer survivors. Psychooncology 22, 54–64. 21905158 10.1002/pon.2047

[14] Denlinger CS & Barsevick AM (2011) The challenges of colorectal cancer survivorship. J Natl Compr Cancer Network 7, 883–894. 10.6004/jnccn.2009.0058 PMC3110673 19755048

[15] Courneya KS, Friedenreich CM, Quinney HA, et al. (2003) A randomized trial of exercise and quality of life in colorectal cancer survivors. Eur J Cancer Care 12, 347–357. 10.1046/j.1365-2354.2003.00437.x 14982314

[16] Cramer H, Pokhrel B, Fester C, et al. (2016) A randomized controlled bicenter trial of yoga for patients with colorectal cancer. Psychooncology 25, 412–420. 26228466 10.1002/pon.3927

[17] Hawkes AL, Chambers SK, Pakenham KI, et al. (2013) Effects of a telephone-delivered multiple health behavior change intervention (CanChange) on health and behavioral outcomes in survivors of colorectal cancer: a randomized controlled trial. J Clin Oncol 31, 2313–2321. 23690410 10.1200/JCO.2012.45.5873

[18] de Vries-ten Have J, Manusama K, Verkaar AJCF, et al. (2024) A randomized controlled intervention trial to study the effect of a personalized lifestyle program on cancer-related fatigue among colorectal cancer survivors: protocol for the SoFiT study. Br J Nutr 1–31. 10.1017/S0007114524001107 38804183

[19] Yellen SB, Cella DF, Webster K, et al. (1997) Measuring fatigue and other anemia-related symptoms with the Functional Assessment of Cancer Therapy (FACT) measurement system. J Pain Symptom Manage 13, 63–74. 9095563 10.1016/s0885-3924(96)00274-6

[20] Van Belle S, Paridaens R, Evers G, et al. (2005) Comparison of proposed diagnostic criteria with FACT-F and VAS for cancer-related fatigue: proposal for use as a screening tool. Supportive Care Cancer 13, 246–254. 10.1007/s00520-004-0734-y 15549424

[21] Burbach JPM, Kurk SA, Coebergh van den Braak RRJ, et al. (2016) Prospective Dutch colorectal cancer cohort: an infrastructure for long-term observational, prognostic, predictive and (randomized) intervention research. Acta Oncol 55, 1273–1280. 27560599 10.1080/0284186X.2016.1189094

[22] Derksen JWG, Vink GR, Elferink MAG, et al. (2021) The Prospective Dutch Colorectal Cancer (PLCRC) cohort: real-world data facilitating research and clinical care. Sci Rep 11, 3923. 33594104 10.1038/s41598-020-79890-y PMC7887218

[23] World Cancer Research Fund/American Institute for Cancer Research (2018) Diet, Nutrition, Physical Activity and Cancer: A Global Perspective. London: World Cancer Research Fund.

[24] Feunekes IJ, Van Staveren WA, Graveland F, et al. (1995) Reproducibility of a semiquantitative food frequency questionnaire to assess the intake of fats and cholesterol in the Netherlands. Int J Food Sci Nutr 46, 117–123. 7621083 10.3109/09637489509012539

[25] Verkleij-hagoort AC, de Vries JH, Stegers MPG, et al. (2007) Validation of the assessment of folate and vitamin B12 intake in women of reproductive age: the method of triads. Eur J Clin Nutr 61, 610–615. 17164826 10.1038/sj.ejcn.1602581

[26] Wendel-Vos GCW, Schuit AJ, Saris WHM, et al. (2003) Reproducibility and relative validity of the short questionnaire to assess health-enhancing physical activity. J Clin Epidemiol 56, 1163–1169. 14680666 10.1016/s0895-4356(03)00220-8

[27] Ainsworth BE, Haskell WL, Herrmann SD, et al. (2011) 2011 compendium of physical activities: a second update of codes and MET values. Med Sci Sports Exerc 43, 1575–1581. 21681120 10.1249/MSS.0b013e31821ece12

[28] Wendel-Vos W, van den Berg S, Duijvestijn M, et al. (2019) Beweegrichtlijnen en Wekelijks Sporter van vragenlijst tot cijfer [Guidelines for Physical Activity and Weekly Sportsman]. Bilthoven. https://www.rivm.nl/bibliotheek/rapporten/2019-0237.pdf (accessed November 2025).

[29] Winkler EAH, Bodicoat DH, Healy GN, et al. (2016) Identifying adults’ valid waking wear time by automated estimation in activPAL data collected with a 24 h wear protocol. Physiol Meas 37, 1653–1668. 27652827 10.1088/0967-3334/37/10/1653

[30] Fisher MI, Cohn JC, Harrington SE, et al. (2022) Screening and assessment of cancer-related fatigue: a clinical practice guideline for health care providers. Phys Ther 102, pzac120. 36179114 10.1093/ptj/pzac120 PMC9525018

[31] Minton O & Stone P (2009) A systematic review of the scales used for the measurement of cancer-related fatigue (CRF). Ann Oncol 20, 17–25. 18678767 10.1093/annonc/mdn537

[32] Ward WL, Hahn EA, Mo F, et al. (1999) Reliability and validity of the functional assessment of cancer therapy-colorectal (FACT-C) quality of life instrument. Qual Life Res 8, 181–195. 10472150 10.1023/a:1008821826499

[33] Kahan BC & Morris TP (2012) Improper analysis of trials randomised using stratified blocks or minimisation. Stat Med 31, 328–340. 22139891 10.1002/sim.4431

[34] Van Buuren S & Groothuis-Oudshoorn K (2011) mice: multivariate imputation by chained equations in R. J Stat Softw 45, 1–67.

[35] Vardy JL, Dhillon HM, Pond GR, et al. (2016) Fatigue in people with localized colorectal cancer who do and do not receive chemotherapy: a longitudinal prospective study. Ann Oncol 27, 1761–1767. 27443634 10.1093/annonc/mdw252 PMC4999562

[36] Ratjen I, Schafmayer C, di Giuseppe R, et al. (2017) Postdiagnostic physical activity, sleep duration, and TV watching and all-cause mortality among long-term colorectal cancer survivors: a prospective cohort study. BMC Cancer 17, Article number 701. 10.1186/s12885-017-3697-3 PMC5657114 29070017

[37] Hoang T, Kim H & Kim J (2020) Dietary intake in association with all-cause mortality and colorectal cancer mortality among colorectal cancer survivors: a systematic review and meta-analysis of prospective studies. Cancers 12, 1–20. 10.3390/cancers12113391 PMC7697273 33207660

[38] van Zutphen M, Kampman E, Giovannucci EL, et al. (2017) Lifestyle after colorectal cancer diagnosis in relation to survival and recurrence: a review of the literature. Curr Colorectal Cancer Rep 13, 370–401. 29104517 10.1007/s11888-017-0386-1 PMC5658451

[39] van Zutphen M, van Duijnhoven FJB, Wesselink E, et al. (2021) Identification of lifestyle behaviors associated with recurrence and survival in colorectal cancer patients using random survival forests. Cancers 13, 2442. 10.3390/cancers13102442 PMC8157840 34069979

[40] Cella D, Eton DT, Lai JS, et al. (2002) Combining anchor and distribution-based methods to derive minimal clinically important differences on the Functional Assessment of Cancer Therapy (FACT) anemia and fatigue scales. J Pain Symptom Manage 24, 547–561. 12551804 10.1016/s0885-3924(02)00529-8

[41] Husson O, Mols F, van de Poll-Franse L, et al. (2015) Variation in fatigue among 6011 (long-term) cancer survivors and a normative population: a study from the population-based PROFILES registry. Supportive Care Cancer 23, 2165–2174. 10.1007/s00520-014-2577-5 25556703

[42] Razak NA, Azhar ZI, Baharuddin IH, et al. (2024) Does exercise improve health-related quality of life of colorectal cancer survivors? A systematic review and meta analysis. Asian Pac J Cancer Prev 25, 379–391. 38415522 10.31557/APJCP.2024.25.2.379 PMC11077098

[43] Vähä-Ypyä H, Sievänen H, Husu P, et al. (2022) How adherence to the updated physical activity guidelines should be assessed with accelerometer? Eur J Public Health 32, I50–5. 36031824 10.1093/eurpub/ckac078 PMC9421411

[44] Mazzoni AS, Nordin K, Berntsen S, et al. (2017) Comparison between logbook-reported and objectively-assessed physical activity and sedentary time in breast cancer patients: an agreement study. BMC Sports Sci Med Rehabil 9, 1–9. 28373907 10.1186/s13102-017-0072-2 PMC5376284

[45] Fazzino TL, Fabian C & Befort CA (2017) Change in physical activity during a weight management intervention for breast cancer survivors: association with weight outcomes. Obesity 25, S109–S115. 29086523 10.1002/oby.22007 PMC5679351

[46] Harrigan M, Cartmel B, Loftfield E, et al. (2016) Randomized trial comparing telephone *v.* in-person weight loss counseling on body composition and circulating biomarkers in women treated for breast cancer: the Lifestyle, Exercise, and Nutrition (LEAN) study. J Clin Oncol 34, 669–676. 26598750 10.1200/JCO.2015.61.6375 PMC4872022

[47] Machado P, Morgado M, Raposo J, et al. (2022) Effectiveness of exercise training on cancer-related fatigue in colorectal cancer survivors: a systematic review and meta-analysis of randomized controlled trials. Supportive Care Cancer 30, 5601–5613. 10.1007/s00520-022-06856-3 35107601

[48] Barsevick AM, Irwin MR, Hinds P, et al. (2013) Recommendations for high-priority research on cancer-related fatigue in children and adults. J Natl Cancer Inst 105, 1432–1440. 24047960 10.1093/jnci/djt242 PMC3888121

[49] Bisschop CNS, Courneya KS, Velthuis MJ, et al. (2015) Control group design, contamination and drop-out in exercise oncology trials: a systematic review. PLoS One 10, e0120996. 25815479 10.1371/journal.pone.0120996 PMC4376879

[50] Smit KC, Derksen JWG, Stellato RK, et al. (2024) Determinants of physical activity among patients with colorectal cancer: from diagnosis to 5 years after diagnosis. Med Sci Sports Exerc 56, 623–634. 38079324 10.1249/MSS.0000000000003351 PMC12376814

[51] van Putten M, Husson O, Mols F, et al. (2016) Correlates of physical activity among colorectal ancer survivors: results from the longitudinal population-based profiles registry. Supportive Care Cancer 24, 573–583. 10.1007/s00520-015-2816-4 PMC4689770 26173977

