# Supplementary material for: The effect of a person-centred lifestyle programme on cancer-related fatigue in colorectal cancer survivors: a randomised trial
Source: Br J Nutr. 2025 Dec 9;135(3):349–60. doi: 10.1017/S0007114525105862 (PMC12912834; doi:10.1017/S0007114525105862)
Supplement: de Vries-ten Have et al. supplementary material [file S0007114525105862sup001.docx]

# Supplementary material

*Table S1. Post hoc analyses in specific subgroups of participants of a randomized study testing the effect of a lifestyle intervention versus control on cancer-related fatigue*

|  | Intervention group | | | Control group | | | Between-group difference^2^ | |
| --- | --- | --- | --- | --- | --- | --- | --- | --- |
|  | n | Baseline^1^ | Six months | n | Baseline | Six months | n | Adj. mean difference (95% CI) |
| Participants with fatigue at baseline | | | | | | | | |
| Fatigue | 61 | 25.3 ± 5.8 | 32.7 ± 9.1 | 59 | 25.2 ± 4.8 | 30.9 ± 7.7 | 120 | 1.7 (-1.1; 4.5) |
| Participants who underwent chemotherapy as part of their treatment | | | | | | | | |
| Fatigue | 36 | 28.8 ± 7.6 | 35.4 ± 9.7 | 38 | 27.6 ± 7.1 | 32.6 ± 8.0 | 74 | 2.3 (-1.6; 6.1) |
| Participants who only had surgery as colorectal cancer treatment | | | |  |  |  |  |  |
| Fatigue | 40 | 27.8 ± 7.4 | 34.0 ± 9.1 | 38 | 29.5 ± 7.1 | 35.0 ± 8.7 | 78 | 0.3 (-3.1; 3.6) |

^1^ means ± standard deviations

^2^The between-group mean difference was adjusted for fatigue at baseline and for the stratification factors used during randomization

*Table S2. Post hoc analyses in specific subgroups of participants of a randomized study testing the effect of a lifestyle intervention versus control on Health-Related Quality of Life (HRQoL)*

|  | Intervention group | | | Control group | | | Between-group difference^2^ | |
| --- | --- | --- | --- | --- | --- | --- | --- | --- |
|  | n | Baseline^1^ | Six months | n | Baseline | Six months | n | Adj. mean difference (95% CI) |
| Participants with fatigue at baseline | | | | | | | | |
| HRQoL total^2^  Physical well-being  Social well-being  Emotional well-being  Functional well-being  Colorectal cancer subscale | 61  61  61  61  61  61 | 93.4 ± 12.6  20.6 ± 3.0  19.4 ± 4.2  17.6 ± 3.7  15.3 ± 4.3  20.5 ± 3.4 | 99.7± 16.1  22.6 ± 3.6  19.7 ± 4.6  19.1 ± 3.7  17.5 ± 5.2  20.9 ± 4.0 | 60  60  60  60  60  60 | 93.3 ± 15.5  20.3 ± 3.1  18.5 ± 5.3  18.2 ± 3.7  15.7 ± 4.3  20.6 ± 4.0 | 98.0 ± 14.4  22.1 ± 3.3  18.7 ± 5.0  19.4 ± 3.4  17.0 ± 4.4  20.9 ± 3.7 | 120  120  120  120  120  120 | 1.6 (-2.2; 5.4)  0.3 (-0.8; 1.3)  0.4 (-0.8; 1.6)  0.0 (-1.0; 1.0)  0.8 (-0.6; 2.2)  0.1 (-1.1; 1.2) |
| Participants who underwent chemotherapy as part of their treatment | | | | | | | | |
| HRQoL total  Physical well-being  Social well-being  Emotional well-being  Functional well-being  Colorectal cancer subscale | 36  36  36  36  36  36 | 95.3 ± 11.5  20.6 ± 2.8  19.5 ± 4.8  18.5 ± 2.8  16.4 ± 3.7  20.4 ± 3.8 | 102.3 ± 14.4  22.3 ± 3.7  19.6 ± 4.8  19.8 ± 3.0  19.0 ± 4.1  21.6 ± 3.7 | 38  38  38  38  38  38 | 95.3 ± 16.6  20.6 ± 3.5  18.8 ± 5.5  18.8 ± 3.5  16.1 ± 3.9  20.9 ± 4.5 | 99.7 ± 16.4  22.0 ± 3.5  19.2 ± 5.1  19.7 ± 2.9  17.7 ± 4.6  21.0 ± 4.4 | 74  74  74  74  74  74 | 2.6 (-3.2; 8.4)  0.2 (-1.5; 1.9)  0.0 (-1.6; 1.5)  0.2 (-1.1; 1.6)  1.1 (-0.7; 2.9)  1.0 (-0.6; 2.5) |
| Participants who only had surgery as colorectal cancer treatment | | | | | | | | |
| HRQoL total  Physical well-being  Social well-being  Emotional well-being  Functional well-being  Colorectal cancer subscale | 40  40  40  40  40  40 | 93.9 ± 14.7  21.7 ± 3.1  18.5 ± 4.9  17.7 ± 3.9  15.4 ± 4.6  20.6 ± 3.5 | 99.1 ± 17.8  23.1 ± 3.6  18.7 ± 4.6  19.0 ± 3.6  16.9 ± 5.5  21.4 ± 4.5 | 38  38  38  38  38  38 | 94.9 ± 14.1  21.5 ± 2.8  18.5 ± 5.5  18.8 ± 3.7  16.2 ± 4.0  20.4 ± 4.0 | 100.7 ± 14.3  23.4 ± 3.1  18.9 ± 5.4  19.5 ± 3.6  17.2 ± 4.2  21.7 ± 3.2 | 78  78  78  78  78  78 | -0.6 (-6.0; 4.7)  -0.4 (-1.7; 0.9)  -0.1 (-1.7; 1.5)  -0.1 (-1.3; 1.1)  0.4 (-1.4; 2.3)  -0.6 (-2.2; 1.0) |

^1^ means ± standard deviations

^2^The between-group mean difference was adjusted for HRQoL at baseline and for the stratification factors used during randomization
